# Studying the Effect of Cold Rolling and Heat Treatment on the Microstructure and Mechanical Properties of the Fe_36_Mn_20_Ni_20_Cr_16_Al_5_Si_3_ High Entropy Alloy

**DOI:** 10.3390/e24081040

**Published:** 2022-07-28

**Authors:** Essam R. I. Mahmoud, Awaludin Shaharoun, Mohamed A. Gepreel, Saad Ebied

**Affiliations:** 1 Department of Mechanical Engineering, Islamic University of Madinah, Madinah 42351, Saudi Arabia; prof.awaluddin@gmail.com; 2 Materials Science and Engineering Department, Egypt-Japan University of Science and Technology, Alexandria 21934, Egypt; mohamed.gepreel@ejust.edu.eg; 3 Department of Production Engineering and Mechanical Design, Faculty of Engineering, Tanta University, Tanta 31527, Egypt; saad_ebied@f-eng.tanta.edu.eg

**Keywords:** high entropy alloys, casting, thermomechanical processing, microstructure, wear resistance

## Abstract

In this study, a multi-component FeMnNiCrAlSi high-entropy alloy, chosen through Thermo-Calc^®^ software (2021a, Stockholm, Sweden) calculation and produced by electric arc melting, was studied for phase continents and mechanical properties. The results elucidated that the cold rolled condition (area reduction ratio about 86%) was in the form of elongated grains with a dendritic structure. Also, small amounts of the BCC phase were precipitated at the grain boundaries. The annealed sample shows features of BCC phase and different sizes of intermetallics. These results coincided with the predictions of Thermo-Calc^®^ software calculations. A cold rolled sample showed high compressive yield strength of about 950 MPa, and the annealed sample had only half the strength of the cold rolled condition. The cold rolled sample shows the highest micro-hardness. The wear resistance of the annealed condition was significantly improved at room temperature and at 200 °C. The brittle phases in the annealed condition have a positive impact on the wear resistance.

## 1. Introduction

High Entropy Alloys (HEAs) provide a new way of developing advanced green materials, because they are recyclable, with unique mechanical and physical properties, which cannot be achieved by conventional alloys [1]. HEAs are considered new members of the metals family, which have established a new paradigm for alloy exploration, which involves the mixing of multiple elements (at least five elements) in an equiatomic or near-equiatomic composition for maximizing compositional entropy [2]. The high mixing entropy will reduce the Gibbs free energy, resulting in solid-solution-phase formation rather than complex intermetallic phases [3]. They exhibit excellent fracture toughness, exceeding that of most conventional alloys [4], outstanding strength at low and high temperatures [5,6], great thermal stability [7], superconductivity [8], significant resistance to corrosion [9], and superparamagnetic, compared to normal, alloys. HEAs have potential for many current and future engineering applications, especially in the transportation and defense industries [10]. Generally, HEAs have many unique features; high entropy of mixing, lattice distortion, closely-spaced nanosized second phase, better efficiency of defect storage, sluggish diffusion kinetics, low-stacking fault energy, and cocktail effects, which give them their properties [11,12].

The phase development depends mainly on the composition of the fundamental elements in the HEAs. So, selecting the elements and their compositions that result in a desirable stable phase is still one of the main parameters that directly affects the HEAs properties. Extensive efforts have been carried out in this area in recent years. One of the methods used to design a new HEA, or in other words, determine the stability of the phase in a new high entropy alloy, is the method of equilibrium thermodynamics, which calculates the different phases of Gibbs free energy that could possibly form in a certain HEA [13,14]. For a specific composition, an estimate of the expected formed phases will be based on which phase will be more energetically advantageous. This is a very difficult and time-consuming process because of the large number of possible HEAs and, consequently, the expected phases. Furthermore, the more elements that are in an alloy, the more difficult the prediction of the generated phases [15]. Another prediction method, called Calculations of Phase Diagrams (CALPHAD), is used to predict expected phases in HEAs. It is a semi-empirical computational approach, which relies on data obtained from binary and ternary diagrams [16]. However, it is possible that the predicted phases from the binary alloys may vary for ternary, quaternary, and high-order alloys.

Many HEAs’ systems were produced for different applications. Senkov et al. [17], developed a series of high-wear resistant HEAs’ light alloys. They melt low-density elements, for example Zr, V, Nb, Ti and Cr, to form a CrNbTiVZr system. Stepanov et al. [18] used the same system but they substituted Zr with Al to manufacture a system of AlCrNbTiV alloy. The resulting alloy shows a higher compressive yield strength, reaching to 1.02 GPa. Youssef et al. [5] developed a novel ultralow density nanocrystalline AlLiMgScTi alloy. It shows a superior strength of 2 GPa, which is several times higher in magnitude than conventional alloys. For high-temperature applications, the CrNbTiVZr HEA system is able to sustain their high strength to a temperature reaching 1200 °C, which is much better than the conventional refractory Inconel 718 superalloy [19,20]. In contrast, for cryogenic temperature applications, Gludovatz et al. [4] developed a CrMnFeCoNi HEA system, which displays superior fracture toughness with excellent tensile strength at a low temperature of −196 °C. Moreover, Nene et al. [21,22] added 1.5 at. % Cu to the FeMnCrCoSi HEA system and found a complete change in phase stability to a stabilized γ-FCC phase. The resultant alloy possesses exceptional properties of ductility, strength, fatigue, and corrosion resistance. Another well-known HEA is the CoCrFeMnNi alloy, also known as the Cantor alloy. This alloy exhibits significant work-hardening capacity and high room- and cryogenic-temperatures’-fracture toughness. However, it has low yield strength, which limits its applications [23]. Ma et al. [24] proved that the Al0.3CoCrFeNi HEA has a lower strain-rate sensitivity and a strong creep resistance. They established that cold rolling can be applied to control the microstructure and hence mechanical properties of different alloys including HEAs [25,26,27,28,29]. Also, cold rolling and sequent annealing change the microstructure and in turn enhance the HEAs’ mechanical properties [30,31,32].

In this work, the effects of cold rolling and heat treatment on the microstructure, mechanical properties and wear resistance of the FeAlNiCrMnSi HEA were investigated. Various characterization methods were applied to characterize the new alloy microstructure, phase composition and tribological properties.

## 2. Experimental Work

The New High Entropy Alloy (HEA) system (Fe_36_Mn_20_Ni_20_Cr_16_Al_5_Si_3_) was designed based on the Thermo-Calc software^®^ calculations from the following common elements of Fe, Mn, Ni, Cr, Al and Si to obtain high strength and good deformability. The alloy was produced using an electric arc furnace (ARCAST 200, Maine, ME, USA) under a high purity argon atmosphere. First, the ingots were produced using high-purity elemental Fe, Mn, Ni, Cr, Al and Si [33]. To ensure full melting and the homogeneity of the ingots, it was melted four times. The ingots were cut into two parts with a rectangular cross section (10.8 mm by 12.66 mm). The two parts were cold rolled, using a Durston rolling mill (FSM 130, Buckinghamshire, UK), to produce two bars with 5 mm diameter (area reduction ratio about 86%), hereinafter called CR.

One bar of the two cold rolled bars was solution-treated (900 °C–30 min), hereinafter called ST. Specimens with a height of 7 mm and diameter of 5 mm were cut and uniaxially compressed at a constant strain rate of 10^−3^ s^−1^ using a Shimadzu UTM (AG-X plus) at room temperature. The pin-on-disk wear apparatus was used for the fabricated alloy tribological properties’ evaluation. The pins were machined to obtain the desired pin shape with a radius of approximately 5 mm. Wear evaluation was quantified as cumulative mass loss of pin through precision electronic weighing balance of 0.1 mg accuracy. Pin samples were rubbed and cleaned before wear-testing. The counter body was #600-grade emery paper (Aluminum oxide particles) fixed on a 304 stainless steel disc. The track radius was 40 mm. The wear test was done with a sliding velocity of 0.6 m/s, and a load of 30 N. The tests were performed at room temperature and at 200 °C. Total sliding time was 20 min. The test was stopped after every 5 min to replace the emery paper and to measure the pin weight. As a reference, rods of the same dimensions made from stainless steel 304 and 316 were subjected to the same conditions. The microstructure investigation was carried out using a scanning electron microscope (SEM) (FESEM/QUANTA FEG) and an optical microscope (Olympus GX71, Buckinghamshire, UK) after electrochemical etching with 10% oxalic acid. X-ray diffraction (D8 Discover with GADDS system, 35 kV, 80 mA) was used in the scanning range of 40 ≤ 2θ ≤ 100° intervals with a step size of 0.05 deg and a scan rate of 1 deg/s to investigate the crystal structure of the cold rolled and solution-treated samples.

## 3. Results and Discussions

### 3.1. ThermoCalc^®^ Analysis and Phase Formation

**Figure 1:**
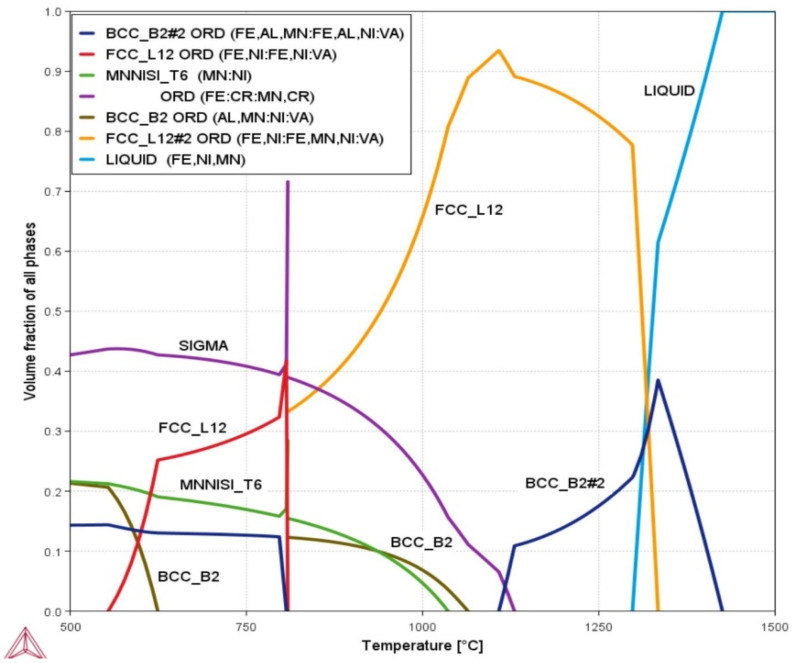
ThermoCalc^®^ calculation equilibrium phase diagram, for Fe_36_Mn_20_Ni_20_Cr_16_Al_5_Si_3_ HEA [33].

**Figure 2:**
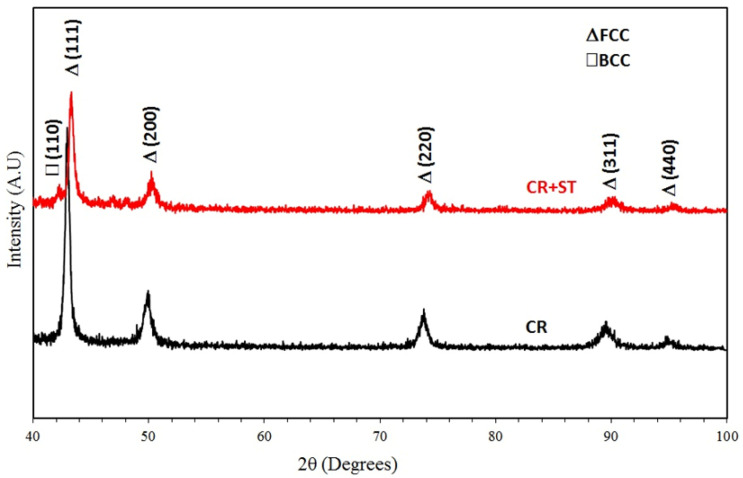
XRD patterns of the CR and CR + ST Fe_36_Mn_20_Ni_20_Cr_16_Al_5_Si_3_ HEA.

The philosophy behind choosing and preparing the Fe_36_Mn_20_Ni_20_Cr_16_Al_5_Si_3_ HEA system was to produce a new low-cost alloy with good properties. Also, the targeted alloy had to be easily deformable for other cost factors (reduced processing cost). Figure 1 demonstrates the equilibrium phase diagram as a function of the temperature performed by ThermoCalc^®^ calculations. The calculations suggested the formation of many phases at room temperature. The structure was composed of a highly deformable FCC phase as the main constituent, which was formed in a temperature range of 550 °C to 1300 °C. This was due to the higher content of Ni and Mn which enhanced the formation of FCC phase, and the lower content of Si and Al, which promoted the formation of other brittle phases. For that reason, these other phases, namely; sigma, silicides, B2 and other intermetallics were present in lower contents and at a lower temperature. The sigma phase can be formed up to about 1120 °C. The BCC-ordered phase (B2) can be formed in a higher short temperature range of 1150 °C to more than 1400 °C in the melt state. This means it is the first solid-phase nucleate from the liquid state. This is due to the high content of the BCC stabilizers such as Fe, Al and Cr. However, the BCC/B2 first to-be-formed phase tends to transform to FCC phase during cooling at high temperature where diffusion is possible. The XRD patterns of the CR and the one followed by CR-ST of the Fe_36_Mn_20_Ni_20_Cr_16_Al_5_Si_3_ alloy are shown in Figure 2. The XRD pattern of the cold rolled condition shows only the FCC phase. This means that the FCC phase started to form during solidification and progressed by retransformation of the BCC/B2 phase to FCC that resulted in a FCC main phase at a low temperature. The precipitation of sigma, B2, or silicides phases from the FCC phase during further cooling at intermediate temperatures below 1000 °C was not possible kinetically, according fully with the ThermoCalc^®^ predicted calculations. The FCC peaks appeared in different orientations and different intensities. On the other hand, the XRD pattern of the CR followed by ST at 900 °C shows mainly the FCC crystal structure with a little BCC/B2 phase structure (presented in low diffraction peak) represented with the (110) peak at 2θ of 42°, as shown as a red color in Figure 2. Holding the CR alloy at 900 °C increases the possibility of BCC phase precipitation where the kinetics allow such precipitation at this temperature. The ST temperature gives a chance for a small amount of the BCC phase to be precipitated from the FCC matrix, which represents the equilibrium state. All the phases predicted by ThermoCalc^®^ calculation appeared in the XRD, except the sigma phase which is kinetically not possible at such treatment conditions. It should be noted that calculations were made on equilibrium conditions for homogeneous alloys and not considering impurities and elemental segregation or any abnormal condition during the casting and solution treatment processes.

### 3.2. Microstructure Characterization

**Figure 3:**
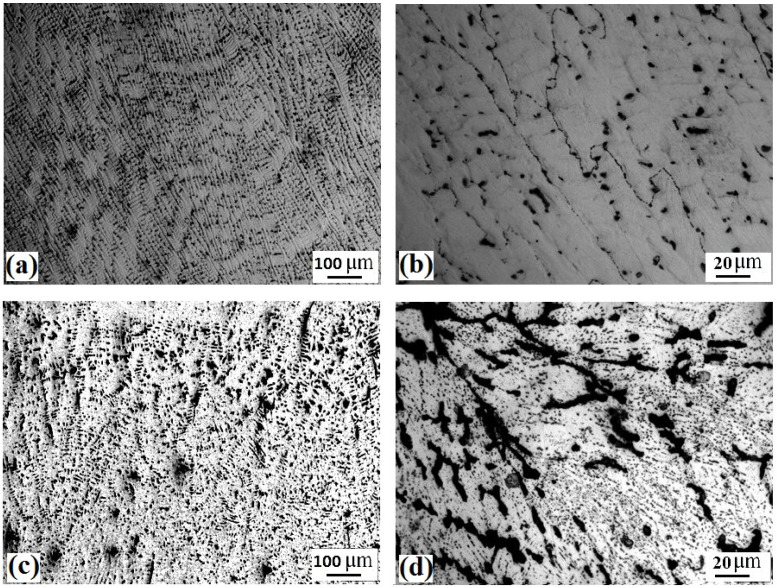
Optical micrographs at different magnifications (high to low from left to right) of the Fe_36_Mn_20_Ni_20_Cr_16_Al_5_Si_3_ HEA in the CR condition (**a**,**b**), and the CR followed by ST condition (**c**,**d**).

The optical microstructure at different areas of Fe_36_Mn_20_Ni_20_Cr_16_Al_5_Si_3_ alloy in the CR and CR-ST conditions are shown in Figure 3. The alloy in cold rolled condition shows a deformed dendritic structure of FCC phase along with dark interdendritic fine zones as shown in low and higher magnification of Figure 3a,b. However, the solution treatment at 900 °C for the cold rolled samples resulted in considerable precipitation of BCC phases (dark phase) in the interdendritic zones. The precipitation of the BCC phase is activated at the interdendritic zones as well as fine intragranular precipitation, as seen in low and higher magnification of Figure 3c,d.

**Figure 4:**
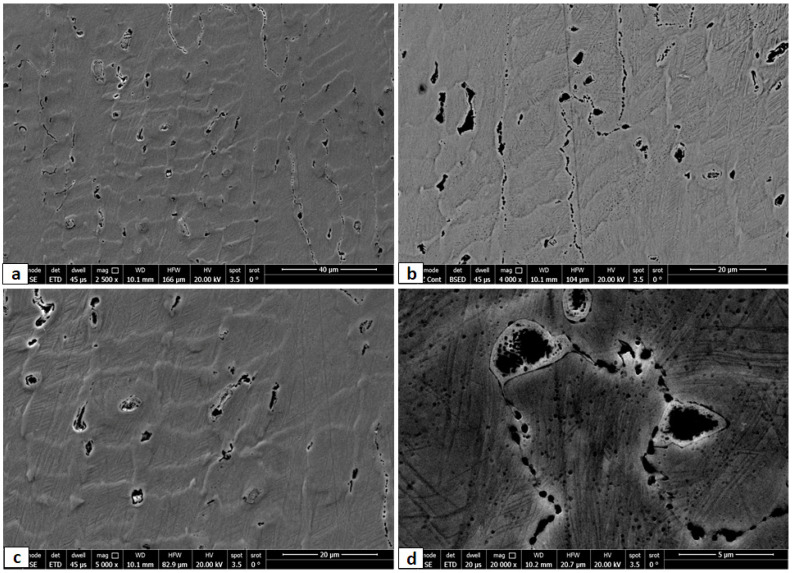
Different magnified SEM images of cold rolled Fe_36_Mn_20_Ni_20_Cr_16_Al_5_Si_3_ HEA at different zones, where (**c**) is higher magnification of (**a**), and (**d**) is higher magnification of (**b**).

**Figure 5:**
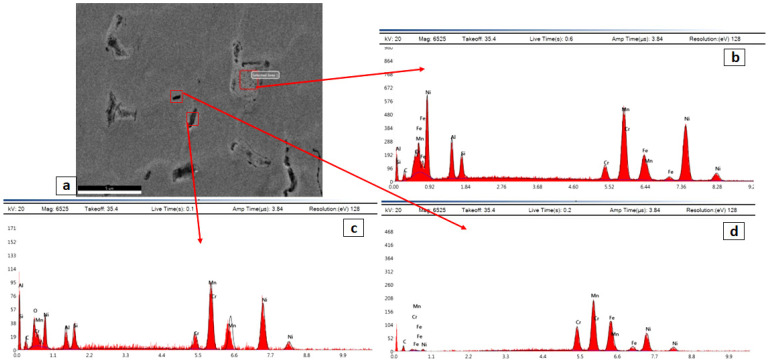
(**b**–**d**) Different EDAS spectra of the red marks in the SEM micrograph (**a**) of cold rolled Fe_36_Mn_20_Ni_20_Cr_16_Al_5_Si_3_ HEA.

A higher magnification of the SEM images in the cold rolled condition are shown in Figure 4. The higher magnification of the CR alloy shows deformation features such as twinning and slip bands. Some intergranular precipitations appeared at the grain boundaries as well as interdendritic zones. These precipitations were analyzed through EDX and the results are shown in Figure 5. The elemental composition of the irregular sized particles at the boundary triple point (Figure 5b) showed higher content of Ni, Al, and Si and lower content of Fe and Cr, which is common for the BCC/B2 phase formed during solidification. The BCC phase and the ordered B2 phase are both reported to be rich with Ni and Al due to the high negative values of ∆Hmix of the Al-Ni [34]. On the other hand, the EDX readings of the FCC matrix show a higher content of Mn, Fe and Ni, as commonly found in such low-cost HEAs [35].

**Figure 6:**
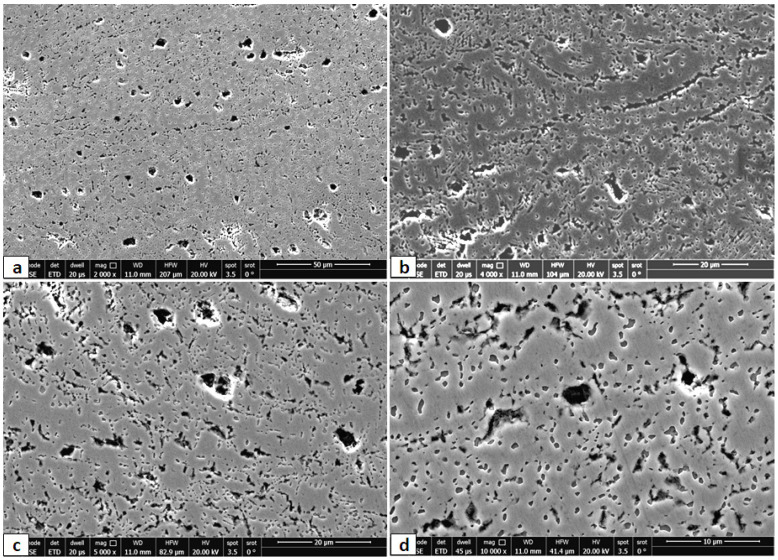
Different magnified SEM micrographs of CR, and ST Fe_36_Mn_20_Ni_20_Cr_16_Al_5_Si_3_ HEA, where (**a**) normal magnification, and (**b**–**d**) higher magnifications of (**a**).

For the solution-treated sample, as shown in Figure 6, many precipitations with different sizes and features appeared as intergranular and transgranular in the matrix. In this annealed condition, the microstructure was homogenous without preferential sites in the grain boundaries of the secondary phases, as was clearly observed in the cold rolled condition. Also, the apparent number of precipitates in the annealed condition is significantly increased in comparison to that of the as-rolled condition, in good agreement with XRD results. Some precipitates appeared as coarse round shapes. Others appeared as very fine-edged particles distributed within the whole matrix. Furthermore, the dendrites structure in the annealed condition were erased during the high-temperature heat treatment. This means that the used temperature and time in the annealed process were sufficient to enable alloying element diffusion to take place.

**Figure 7:**
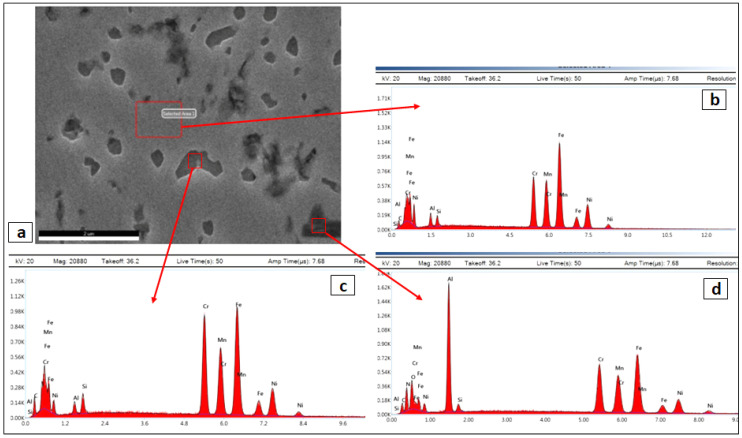
(**b**–**d**) Different EDAS spectra of the red marks in the SEM micrograph (**a**) of CR and ST Fe_36_Mn_20_Ni_20_Cr_16_Al_5_Si_3_ HEA.

To confirm the nature and type of these precipitates, EDX analysis was performed, and the results were shown in Figure 7. The EDX of the FCC matrix as shown in Figure 7b presented peaks of Al, Si, Cr, Mn, Fe and Ni with atomic fractions of 4, 3, 15, 16, 34 and 16, respectively, which is very close to the alloy composition. These compositions represent the FCC common phase. The edged precipitate, shown in Figure 7c, showed higher concentrations of Cr, Si and Al (27, 15 and 8 wt.%, respectively), which most probably represent the BCC/B2 phase which is indexed by XRD. The black irregular shape precipitates represented in Figure 7d showed a very high content of Al and Cr in addition to lower content of Fe and Mn. There may be few metastable Al-silicides formed by precipitation during the annealing process.

### 3.3. Mechanical Properties

**Figure 8:**
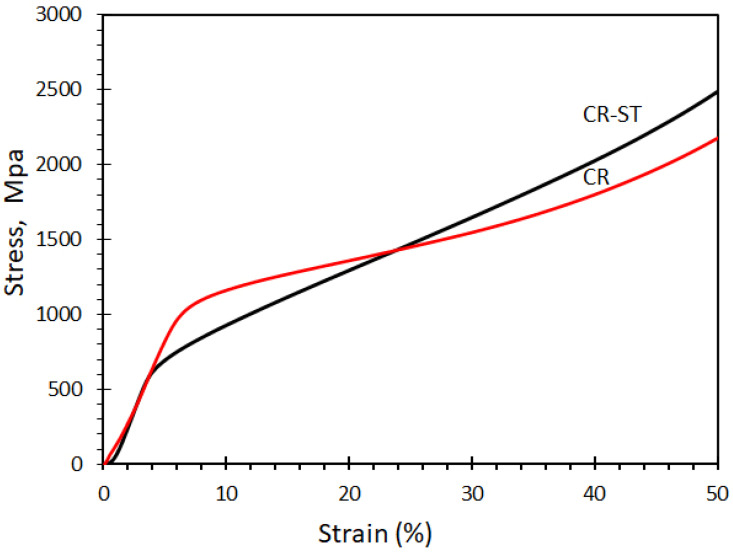
Engineering stress-strain curve of Fe_36_Mn_20_Ni_20_Cr_16_Al_5_Si_3_ HEA at room temperature.

The compressive engineering stress–strain curve of the alloy in the CR and CR-ST conditions is shown in Figure 8. The compressive yield strength is about 550 MPa for the ST condition which is low compared to about 950 MPa for the CR condition. These could be related to the high dislocation density caused by the cold rolling action (i.e., a result of work-hardening by cold rolling of ~85% reduction in area) of the FCC matrix. Also, annealing treatment resulted in hardness softening as it decreased from 240 ± 5 HV for the as-rolled condition to 208 ± 5 HV for the annealed state. This is due to the great differences in the microstructure. However, the CR-ST alloy showed a higher work hardening rate than the CR condition. This is related to starting microstructure where the CR alloy consists of mainly the FCC phase while CR-ST consists of mainly the FCC phase besides the minor BCC/B2 precipitates. The multiphase ST alloy would harden more by deformation at room temperature.

**Figure 9:**
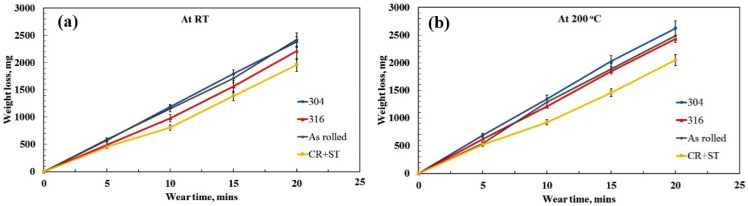
Wear rate of Fe_36_Mn_20_Ni_20_Cr_16_Al_5_Si_3_ HEA in comparison with stainless-steel grades 304 and 316 alloys as a function in the wear time at (**a**) Room temperature (**b**) 200 °C.

Figure 9 shows the weight losses with the sliding time at different temperatures (room temperature and 200 °C) during the ball-on-disc sliding test for the Fe_36_Mn_20_Ni_20_Cr1_6_Al_5_Si_3_ alloy at its as-rolled and as-annealed treated conditions. Conventional stainless-steel alloys of 304 and 316 samples with the same dimensions were subjected to similar testing conditions for comparison. The annealed heat-treated alloy at room temperature shows the lowest weight losses among the tested samples. It was better than both the 316 and 304 samples. This is due to the presence of a hard homogenous BCC/B2 phase that precipitated during the annealing process and acted as obstacles and prevented the wear losses. Moreover, the silicates that are found in the high entropy alloy offer self-lubricating properties to the worn surface, which reduce the weight losses. The wear resistance of the as-rolled condition sample lies between the 304 and 316 samples. This is due to the high density of dislocations and residual stresses that are created during the rolling processes. At a higher temperature of 200 °C, the average weight losses were slightly increased for all the samples due to the possible oxidation. The same trend was observed where the new HEA in the CR-ST condition showed the best wear resistance compared to the commercial 304 and 316 grades of stainless steel. The easy oxidized elements such as Al and Si have lower content, although the elements that have higher content such as Fe, Mn and Cr need more temperature and time to oxidize. The Cr, Si, and Al rich BCC phase seems favorable to improve the wear resistance of the HEA at room and 200 °C temperatures.

## 4. Conclusions

A novel high-entropy Fe_36_Mn_20_Ni_20_Cr_16_Al_5_Si_3_ alloy was designed using ThermoCalc^®^ software and casted using electric arc furnace. The fabricated alloy was cold rolled and annealed at 900 °C for 30 min. The microstructure and mechanical properties of the alloy in its as-rolled and in annealed conditions were investigated. The key findings of this study were:

- The as-rolled Fe_36_Mn_20_Ni_20_Cr_16_Al_5_Si_3_ HEA was composed of an elongated FCC phase with evident dendritic structure. Very few amounts of the BCC phase were precipitated in the grain boundaries. These experimentally observed phases are in good agreement with the predictions of ThermoCalc^®^ software.
- By applying annealing treatment to the as-rolled Fe_36_Mn_20_Ni_20_Cr_16_Al_5_Si_3_ HEA, different sizes and features of BCC phases and intermetallics were found in higher ratio throughout the structure.
- Higher compressive yield strength of about 950 MPa was achieved in the as-rolled condition, although the annealed sample lost almost half of its strength.
- The wear resistance of the annealed condition was significantly improved at room and higher temperature. The brittle phases in the annealed condition were found to have a positive impact on the wear resistance.

## Data Availability

Not applicable.
